# Comparison of Two Doses of Elemental Iron in the Treatment of Latent Iron Deficiency: Efficacy, Side Effects and Blinding Capabilities

**DOI:** 10.3390/nu6041394

**Published:** 2014-04-04

**Authors:** Alecia J. Leonard, Kerry A. Chalmers, Clare E. Collins, Amanda J. Patterson

**Affiliations:** 1 Priority Research Centre in Physical Activity and Nutrition and School of Health Sciences, Faculty of Health and Medicine, University of Newcastle, Callaghan, NSW 2308, Australia; E-Mails: Alecia.Leonard@uon.ed.au (A.J.L.); Clare.Collins@newcastle.edu.au (C.E.C.); Amanda.Patterson@newcastle.edu.au (A.J.P.); 2 School of Psychology, Faculty of Science and IT, University of Newcastle, Callaghan, NSW 2308, Australia

**Keywords:** ferrous sulfate, side effects, women, serum ferritin

## Abstract

Adherence to iron supplementation can be compromised due to side effects, and these limit blinding in studies of iron deficiency. No studies have reported an efficacious iron dose that allows participants to remain blinded. This pilot study aimed to determine a ferrous sulfate dose that improves iron stores, while minimising side effects and enabling blinding. A double-blinded RCT was conducted in 32 women (18–35 years): 24 with latent iron deficiency (serum ferritin < 20 µg/L) and 8 iron sufficient controls. Participants with latent iron deficiency were randomised to 60 mg or 80 mg elemental iron or to placebo, for 16 weeks. The iron sufficient control group took placebo. Treatment groups (60 mg *n* = 7 and 80 mg *n* = 6) had significantly higher ferritin change scores than placebo groups (iron deficient *n* = 5 and iron sufficient *n* = 6), *F*(1, 23) = 8.46, *p* ≤ 0.01. Of the 24 who completed the trial, 10 participants (77%) on iron reported side effects, compared with 5 (45%) on placebo, but there were no differences in side effects (*p* = 0.29), or compliance (*p* = 0.60) between iron groups. Nine (69%) participants on iron, and 11 (56%) on placebo correctly guessed their treatment allocation. Both iron doses were equally effective in normalising ferritin levels. Although reported side-effects were similar for both groups, a majority of participants correctly guessed their treatment group.

## 1. Introduction

Young women are at high risk of iron deficiency secondary to menstruation and childbirth [1]. The nutritional disorder affects one in five young women in Australia [2] and is associated with poorer general health and wellbeing and high levels of fatigue [3,4]. It is imperative that iron deficiency is effectively managed to prevent progression to anaemia. Increased dietary iron intake, iron fortification and iron supplementation are used to improve iron status [5]. Clinical practice guidelines for the management of iron deficiency have been developed in the United States [6], the United Kingdom [7] and in Australia [8,9]. These all recommend the use of dried ferrous sulfate which contains approximately 33% elemental iron. Clinical practice guidelines recommend a daily dose of 80–105 mg of elemental iron for treatment of iron deficiency anaemia in adults [10]. A systematic review conducted in 2011 assessed the effects of intermittent oral iron supplementation on anaemia in menstruating women, compared with no intervention, a placebo or daily supplementation [11]. This study found weekly supplementation with 60 to 120 mg elemental iron was effective in improving haematological markers [11]. Treatment of latent iron deficiency and the impact of using lower dose iron treatment on iron status are not articulated within current literature and iron treatment guidelines.

Ideally, supplementation should achieve maximal absorption with minimal side effects [12]. Oral iron has been associated with gastrointestinal side effects such as nausea, constipation and darkening of stools which can decrease compliance [8,13]. Such side effects can compromise blinding within trials. Lower dose iron supplements have fewer side effects [10,14] yet the effect of varying the dosage of iron on iron status [15,16,17] has rarely been studied, with no studies conducted in non-pregnant young women. Whether lower doses are absorbed as efficiently as higher doses in non-pregnant young women remains unknown [18]. Therefore, the current study aims to determine the efficacy of two different doses of iron supplementation in improving iron status whilst maintaining blinding to treatment groups.

## 2. Experimental Section

Testing was conducted at the University of Newcastle, Callaghan Campus in NSW, Australia between April 2010 and April 2013. Women aged 18–35 years were recruited via flyers and promotion in lectures within the University. Recruitment also included flyers at the Technical Education (TAFE) College, accessing the volunteer register at Hunter Medical Research Institute and word-of-mouth. All interested individuals were screened for eligibility against inclusion criteria using an author designed questionnaire (refer to supplementary material). The inclusion criteria were: female, 18–35 years; BMI 18–30 kg/m^2^; English as primary language; not iron deficient within the last 12 months; not currently taking iron supplementation (those who had been on a standard multivitamin, containing minimal or no iron, were eligible to participate and asked to cease the supplement); no chronic medical condition; not taking medication that could potentially interfere with results; ability to provide blood samples for biomarkers of iron status; not having donated blood within the last three months and will not donate blood during the trial; not pregnant, or planning a pregnancy within the following 4 months; available to participate in intervention for 4 months. Those eligible were provided with an information statement and informed consent was obtained prior to the commencement of the study. 

### 2.1. Participants

**Figure 1:**
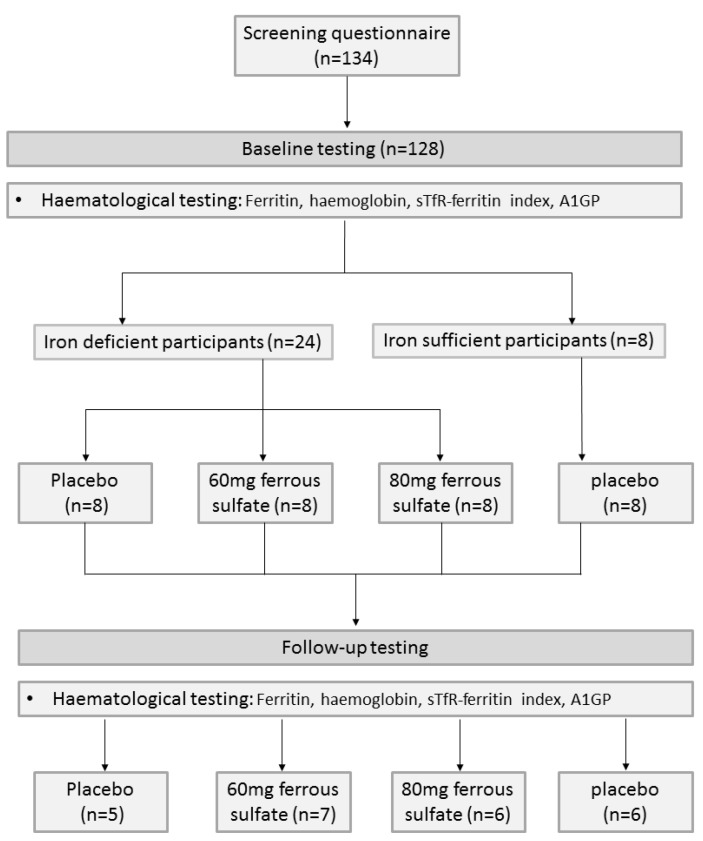
Study recruitment and randomisation flow chart.

Abbreviations: sTfR; soluble transferrin receptor, A1GP; Alpha-1-Glycoprotein.

Thirty two women were included in the intervention. As shown in Figure 1, eight participants were included in the iron sufficient control group and were provided placebo capsules, and 24 iron deficient participants were randomised to either placebo, or treatment (60 mg or 80 mg iron).

### 2.2. Haematological Testing

Serum ferritin, haemoglobin and soluble transferrin receptor (sTfR) were used as biomarkers of iron status, and alpha-1glycoprotein (A1GP) was used as a marker of inflammation. A1GP is slower to rise, but remains at a high concentration longer than C-reactive protein (CRP), so is a better indicator of chronic sub-clinical infection than CRP, and may better reflect changes in the concentration of ferritin during infections [1]. Blood tests were performed by Hunter Area Pathology Service, accredited by the National Association of Testing Authorities Australia, using standard techniques. The timing of the blood testing was not restricted in order to optimise recruitment and compliance [19]. Results of the blood tests were sent directly to the research team at the University, and participants remained blinded to blood test results until the completion of the trial. Iron deficiency was defined as having ferritin < 20 µg/L [20] and all other markers within reference ranges (haemoglobin 115–165 g/L [20], soluble transferrin receptor 0.9–2.30 mg/L [21,22], A1GP 0.51–1.17 g/L [23]). sTfR reflects the number of iron receptors expressed on cell membranes and is raised once tissue iron starts to become limited [24]. It should theoretically represent a definitive marker of latent iron deficiency [25]. Participants with haemoglobin results below the reference range were excluded from the intervention and were immediately referred to their General Practitioner. At completion of the trial, all women were given copies of their blood test results for communication with their General Practitioner.

### 2.3. Pilot Testing of Supplementation

Figure 1 summarises group allocation and progression through the trial. Young women found to be iron deficient at baseline were randomly assigned to one of two different doses (60 mg or 80 mg) of ferrous sulfate or placebo for 16 weeks. Ferrous sulfate was used as it is the most common type of elemental iron used to treat iron deficiency; two different doses were used to determine the most effective dose in improving iron status with the fewest side-effects. The doses 60 mg and 80 mg have been associated with fewer side-effects than the doses recommended in the National Guidelines [10,14]. The duration of supplementation was chosen based upon the correction of iron deficiency anaemia taking between 2 and 4 months [10]. The first eight iron sufficient participants were invited into the intervention as a control group. A single blinding approach was used with the control group who were all provided with placebo capsules, which contained Lactose. Subsequent iron sufficient participants exited the study following baseline testing. Participants were not informed of their treatment or iron status until trial completion. All participants were contacted on a four weekly basis to report any potential side-effects, using a specifically designed questionnaire. It was explained to participants that any remaining capsules would be counted following the intervention to increase compliance. In addition, participants were provided with a “Tips and Reminders” sheet for taking capsules. This included the following information: Take one capsule per day; Leave your container of capsules next to your tooth brush; Keep one or two capsules in the small container provided and leave this in your handbag in case you forget to take it in the morning and then remember part way through the day; Use the calendar to cross off each day once you have taken your capsule. This will help you to keep a track of how regularly you are remembering to take them; Take the capsule two hours apart from any other regular medication (except the oral contraceptive pill which can be taken at the same time); Do not take two capsules on the same day to compensate for missing your capsule the previous day—Take only one capsule per day; Return any left-over capsules in your container when you return for your follow up testing; Please take note of your compliance with the treatment regimen and any side effects you may have experienced and report this to the research team when you are contacted by phone every four weeks. Immediately following the 16 week intervention participants were asked to guess which treatment protocol they thought they had been allocated to. Participants had repeat blood tests after 16 weeks.

### 2.4. Capsules and Randomisation

Compounding chemists were contracted to provide the iron and placebo supplements and used Random Allocation Software to allocate treatments to participant Identification Numbers [26]. The active and placebo supplements were identical in appearance and were packaged in identical containers. The researchers and participants remained blinded to the treatment protocol and the randomisation code was held by a third party researcher only to be broken once the final results were collected. The study protocol was approved by the University of Newcastle Human Research Ethics Committee. 

### 2.5. Statistical Analysis

STATA-IC 11 statistical analysis software was used with an alpha level of 0.05 set for statistical significance. Kruskal-Wallis rank test was used to analyse the effect of group on iron markers at baseline and follow-up and the difference in ferritin change score between oral contraceptive pill users and non-users on iron treatment. One-way analysis of variance (ANOVA) was used to examine the difference in iron marker change scores between treatment (60 mg iron and 80 mg iron) and no treatment groups (control and placebo). Fischer’s exact test was used to examine the frequency of reported side effects and the frequency of correct treatment guesses between treatment and no treatment groups.

## 3. Results

### 3.1. Participants

Twenty-four (75%) participants (mean age ± SD 25.6 ± 4.1 years) completed the intervention (60 mg iron *n* = 7, 80 mg iron *n* = 6, placebo *n* = 5, control *n* = 6). Reasons given for withdrawing from the study were unrelated illness (*n* = 3) or being too busy (*n* = 3). Two participants gave no reason. Participant demographics are shown in Table 1. Participants were primarily Australian, had a mean BMI of 21.2 kg/m^2^ and 48% used an oral contraceptive pill (OCP).

**Table 1:** Participant demographics (*n* = 24).

	60 mg iron	80 mg iron	Placebo	Control
Age (years)	27.9 ± 5.1	24.5 ± 3.4	24.8 ± 3.8	24.7 ± 3.7
BMI (kg/m^2^)	20.8 ± 1.7	21.7 ± 1.2	21.9 ± 2.4	20.6 ± 2.0
Origin				
Australia	4	3	5	2
Asia	1	0	0	1
Canada	1	0	0	0
United Kingdom	0	1	0	0
OCP use (total)	3	3	4	2

Note: BMI: Body mass index, Age and BMI data is provided as mean ± SD.

### 3.2. Iron Status

Ferritin, haemoglobin, and sTfR levels at baseline and follow-up, together with change scores for each group (60 mg iron, 80 mg iron, iron deficient placebo, iron sufficient control) are presented in Table 2. The A1GP was normal in all participants and was unchanged following the intervention. 

**Table 2:** Mean (±SEM) haematological markers of iron status at baseline, follow-up and change scores by treatment group.

Iron marker	60 mg iron	80 mg iron	Placebo	Control
Ferritin (µg/L)				
Baseline	11.1 ± 1.9	10.5 ± 1.7	13.5 ± 2.1	30.4 ± 2.9
Follow-up	34.4 ± 10.2	30.7 ± 7.0	15.1 ± 1.8	31.9 ± 5.0
Change	23.3 ± 10.6	20.3 ± 5.6	1.6 ± 2.0	1.5 ± 4.8
Haemoglobin (g/L)				
Baseline	125.8 ± 3.7	133.7 ± 2.0	132.2 ± 3.3	126.8 ± 1.9
Follow-up	130.1 ± 2.3	136.3 ± 4.2	131.6 ± 3.9	129.0 ± 4.6
Change	4.3 ± 4.0	2.7 ± 3.3	−0.6 ± 2.3	2.2 ± 3.6
sTfR-index				
Baseline	1.4 ± 0.3	1.2 ± 0.3	1.1 ± 0.2	0.7 ± 0.6
Follow-up	0.8 ± 0.8	0.7 ± 0.1	0.9 ± 0.1	0.6 ± 0.9
Change	−0.3 ± 0.2	−0.6 ± 0.2	−0.18 ± 0.1	−0.0 ± 0.1

Note: 60 mg and 80 mg iron: ferrous sulfate, sTfR-Index: soluble transferrin receptor-ferritin index. Normal ranges for haematological markers: ferritin > 20 µg/L; haemoglobin 115–165 g/L; soluble transferrin receptor 0.9–2.30 mg/L.

#### 3.2.1. Baseline

Kruskal-Wallis analyses performed on iron status markers for the three iron deficient groups (60 mg, 80 mg and placebo) confirmed there were no significant between group differences in ferritin (*p* = 0.38), haemoglobin (*p* = 0.34) or sTfR-Index (*p* = 0.82) at baseline. As shown in Table 3, analyses comparing iron sufficient (controls) and iron deficient participants (60 mg, 80 mg and placebo groups combined) revealed that controls had significantly higher ferritin (*p* < 0.01) and lower sTfR-Index (*p* < 0.01) than the combined iron deficient groups at baseline, but no difference in haemoglobin (*p* = 0.30).

**Table 3:** Comparison of haematological markers at baseline, follow-up and change scores.

Comparisons (*p* value)	Ferritin	Haemoglobin	sTfR-Index
Baseline			
Controls *vs.* Iron deficient	<0.01	0.30	<0.01
Follow-up			
Placebo *vs*. Controls, 60 mg, 80 mg	<0.01	1.0	0.11
Change score			
Iron treatment *vs.* Placebo	<0.01	0.45	0.07

#### 3.2.2. Follow-Up

Analysis of iron status at follow-up revealed a significant difference between the placebo group *vs.* the 60 mg, 80 mg and iron sufficient controls combined in ferritin (*p* ≤ 0.01), but no difference in haemoglobin (*p* = 1.0) or sTfR-Index (*p* = 0.11) (as shown in Table 3). Post hoc analysis showed the placebo group had significantly lower ferritin at follow-up than the 60 mg iron group, 80 mg iron group and controls (*p* = 0.02, *p* = 0.02, *p* = 0.04). There was no significant difference in ferritin between controls and 60 mg iron (*p* = 0.57), controls and 80 mg iron (*p* = 0.87), or 60 mg and 80 mg groups (*p* = 0.89) at follow-up.

#### 3.2.3. Change Scores

Change scores between baseline and follow-up for the iron treatment (60 mg and 80 mg combined) and placebo (iron deficient placebo and iron sufficient controls combined) groups were compared using one-way ANOVA. As shown in Table 3, the analyses revealed that the increase in ferritin levels was significantly greater following iron treatment compared with placebo, *F*(1, 23) = 8.46, *p* ≤ 0.01. There were no differences in haemoglobin change, *F*(1, 22) = 0.60, *p* = 0.45, or sTfR-Index change, *F*(1, 15) = 3.95, *p* = 0.07, between iron treatment and placebo groups.

Based on our criteria for iron deficiency (ferritin < 20 µg/L, haemoglobin 115–165 g/L, soluble transferrin receptor 0.9–2.30 mg/L, A1GP 0.51–1.17 g/L), at follow-up six (75%) iron deficient participants on 60 mg iron became iron sufficient and one remained iron deficient (13%). Four iron deficient (57%) participants on 80 mg iron became iron sufficient and two remained iron deficient (28%). Two iron deficient (25%) participants on placebo become iron sufficient and four (50%) remained iron deficient. All except one (who became iron deficient) of the iron sufficient controls (80%) remained iron sufficient (see Table 4). Of the participants on iron treatment, there was no difference in ferritin change score between oral contraceptive pill users and non-users (*p* = 0.94).

**Table 4:** Iron status outcome, compliance, side effects and treatment guesses by treatment group.

Participant group	Outcome			Compliance (%) *	Side effects					Treatment guess		
	IS	ID	DNF		Nil	Nausea	Dark stools	Constipation	Diarrhoea	Iron	Placebo	Unsure
60 mg iron (*n* = 7)	6	1	1	85.7 ± 17.7	5	1	1	0	0	6	1	0
80 mg iron (*n* = 6)	4	2	2	93.3 ± 10.6	2	1	4	1	2	3	2	1
ID placebo (*n* = 5)	1	4	3	92.3 ± 5.3	4	1	0	0	2	2	1	2
IS controls (*n* = 6)	5	1	2	88.7 ± 9.1	5	1	1	0	0	1	5	0

60 mg and 80 mg iron: ferrous sulfate, ID: iron deficient; IS: iron sufficient; DNF: Did not finish; * Compliance is based on the percentage of capsules participants returned at the end of the 16 weeks trial period out of a maximum of 112.

### 3.3. Side Effects and Compliance

Reported side effects included nausea, darkening of stools and constipation. While these were more commonly reported by participants in the 80 mg elemental iron group, particularly dark stools (see Table 4), a Fischer’s exact test indicated there was no statistically significant differences in the frequency of reported side effects between the 60 mg and 80 mg groups (*p* = 0.29), the placebo and controls (*p* = 0.55), or between the treatment and placebo groups (*p* = 0.42). Kruskal-Wallis analyses performed on compliance scores (% of capsules taken) showed no statistically significant difference between the 60 mg and 80 mg groups (*p* = 0.22), between the placebo and controls (*p* = 0.25), or between the treatment and placebo groups (*p* = 0.60).

### 3.4. Participants’ Treatment Guesses

Of the 13 participants taking iron supplements who completed the trial, 9 (69%) correctly guessed they were taking iron supplements. A Fischer’s exact test showed no difference in the number of correct treatment guesses between the 60 mg and 80 mg groups (*p* = 0.27). Of the 11 participants taking placebo capsules who completed the trial, 6 participants (56%) correctly guessed that they were taking placebo capsules. There was also no significant difference in the number of correct treatment guesses between the placebo and control groups (*p* = 0.08).

## 4. Discussion

### 4.1. Change in Iron Status

Limited knowledge exists on the efficacy of different doses of iron supplementation on iron status in non-pregnant young women with latent iron deficiency. Our study aimed to determine a ferrous sulfate dose that improves iron stores in women with latent iron deficiency, while minimising side effects. At follow-up, iron deficient participants who were randomised to ferrous sulfate (60 mg or 80 mg) had significant improvements in their ferritin from baseline levels. Following this improvement, there was no difference in ferritin when compared to controls at follow-up. Iron deficient participants randomised to placebo had significantly lower ferritin than iron treatment groups and controls at follow-up. This shows that 16 weeks of elemental iron is effective in normalising iron levels in most participants in this population of young women, and that without such iron treatment iron stores remain depleted. This pilot study had 68% power to detect a difference in ferritin change score between treatment or no treatment groups. The analysis showed a significantly higher ferritin change in treatment compared to no treatment groups. The power in this study will be used to inform the sample size in future studies as data on treatment of latent iron deficiency in non-pregnant women is limited.

Ferritin increased in iron deficient participants on placebo to a much lesser degree than those on iron treatment. Altering dietary iron was not part of the intervention and participants were not given any advice about changing their diet in order to keep their current intakes stable during the trial. It is possible that participants altered their dietary iron intake, which may explain some of the change in iron status and is a limitation that must be acknowledged. However, participants remained blinded to their iron status until the completion of the trial, so it is just as likely that iron sufficient controls changed their dietary iron intake as the iron deficient participants. Results also demonstrated that a daily 60 mg dose was as effective as an 80 mg dose in treating latent iron deficiency. Seventy five per cent of participants on 60 mg iron dose became iron sufficient at the end of the trial as compared with 57% of participants in the 80 mg iron group. However, there was no significant difference in iron status at follow-up between the 60 mg *vs.* 80 mg iron groups.

A systematic review of the literature has actually shown that weekly dosing at 60–120 mg is adequate for treating iron deficiency in menstruating women [11], however national guidelines recommend 80 or 105 mg daily, which is also recommended by General Practitioners in Australia [10]. We have shown that 60 mg daily is efficacious in young women with latent iron deficiency.

### 4.2. Compliance

The incidence of reported side effects was not statistically significantly different between placebo or treatment groups in this trial. The gastrointestinal effects of iron supplementation appear to be highly individual. Clear dose related side effects have been reported in previous studies using low (15 mg) and high doses (222 mg) [8,15], whereas others have found no difference in side effects between placebo and treatment groups, even when daily doses of 260 mg were used [27]. In the current study, there was no statistically significant difference in the compliance between groups. Galloway *et al*. (1994) reviewed literature on participants’ compliance with iron supplement regimes in research studies and reported that compliance decreases when dose increases, however, as in the current study, Galloway found little evidence of side effects causing low compliance [28].

### 4.3. Side Effects and Treatment Guess

This study also aimed to examine the effect of potential side effects of the two different doses of iron supplementation on awareness of blinding to treatment groups. To assist with blinding, capsules were used in the study rather than tablets. This is due to ferrous sulfate being slightly green in colour and having a distinctly metallic taste. Therefore, to produce tablets for a blinded trial would involve finding inactive compounds to mimic or hide both the colour and taste of ferrous sulfate. Seventy seven per cent of participants in the treatment groups could guess that they were on iron, which is much higher than the 48% of 191 correctly guessing they were taking iron reported by Makrides *et al.* [16], though this study was in pregnant women who were obviously undergoing significant bodily changes making any additional effects of iron treatment difficult to identify. In the current study, the incidence of reported side effects was not different between treatment groups and placebo. This suggests that factors other than side effects play a role in the identification of their treatment, such as perhaps feeling more energetic. Although there was no formal assessment of fatigue and vitality in the current study, Patterson *et al.* (2001) showed improved vitality and decreased fatigue after treatment of iron deficiency in young women [4].

### 4.4. Limitations

Several limitations of this study must be acknowledged. These include the small sample size, and low power, which are likely to have affected the reliability of results. Some participants may have self-selected for this study given that they thought they were iron deficient, however, we made it clear that participants with iron deficiency within the 12 months prior to their enrolment in the study were not eligible. Also, physical activity and dietary intake were not assessed. These factors may have influenced individuals iron status at follow-up [29]. Despite possible influence of day-to-day variation [30], menstruation [31] and seasonal variation [32] on hematological results, the timing of the blood testing was not controlled to prevent unnecessary increased participant burden.

## 5. Conclusions

Results of this study revealed that a 60 mg iron dose can normalize iron status in non-pregnant young women with latent iron deficiency. No differences were found in the incidence of reported side effects or the level of compliance between treatment groups and placebo. Further double-blinded trials should examine the effectiveness of iron doses lower than 60 mg for improving iron status in young women, and to determine if awareness of treatment allocation is reduced.
